# Incidence of hospital-acquired toxin-producing clostridioides difficile infection between the pre-pandemic (2017–2019) and pandemic (2020–2022): a retrospective cohort study

**DOI:** 10.1186/s13690-026-01859-6

**Published:** 2026-02-20

**Authors:** Raquel García Rodríguez, María José Pereira Rodríguez, Alejandra Pilar García López, Fabián Freijedo Fariñas, Angela Nogueira Gómez

**Affiliations:** Preventive Medicine and Public Health Service, A Coruña University Hospital, As Xubias 84, 15006 Spain

**Keywords:** Clostridioides difficile, Epidemiology, Infection prevention and control, Health care associated infection

## Abstract

**Background:**

In 2020, the World Health Organization declared SARS-CoV-2 a global public health emergency. Healthcare systems were forced to reorganize care delivery and implement wide-ranging infection control strategies. Among hospital-acquired infections, toxin-producing *Clostridioides difficile* infection remains a major concern due to its transmission via contact and its association with high morbidity and mortality. Although primarily aimed at preventing viral transmission, the measures introduced during the COVID-19 pandemic may have influenced the incidence of other nosocomial infections, including toxin-producing *Clostridioides difficile* infection.

This study aimed to evaluate and compare the incidence of nosocomial toxin-producing *Clostridioides difficile* infection during the pandemic and pre-pandemic periods, and to confirm associated risk factors across both periods.

**Methods:**

We conducted a retrospective observational cohort study at A Coruña University Hospital, including data from 2017 to 2022. Patients meeting criteria for nosocomial *Clostridioides difficile* infection were categorized into pre-pandemic (2017-2019) or pandemic (2020-2022) cohorts. Variables analyzed included demographics (age, sex), prior antibiotic use, antiulcer therapy, immunosuppression and surgical history. Incidence rates were calculated and compared between periods, and associations between risk factors and toxin-producing *Clostridioides difficile* infection were analyzed using odds ratios (OR).

**Results:**

A total of 249 nosocomial toxin-producing *Clostridioides difficile* infection cases were identified: 89 pre-pandemic and 160 during the pandemic, reflecting a 79.8% increase. Patients hospitalized during the pandemic faced a 92% greater risk of toxin-producing *Clostridioides difficile* infection (RR=1.92; CI95%: 1.48-2.49; *P*=0.001). Established risk factors such as prior antibiotic exposure (80% during the pandemic vs 86.5% pre-pandemic; *P*=0.20) and immunosuppression was frequent (39.3% pre-pandemic; 46.9% pandemic; *P*=0.25).

Notably, the use of proton pump inhibitors significantly increased during the pandemic (*P*=0.02; 95%CI: 0.02–0.36). Surgical history, particularly gastrointestinal surgery, was significantly associated with complications (OR=6.6, 95%CI: 2.18-20.18). The incidence density of toxin-producing *Clostridioides difficile* infection (TCDI) during the pandemic years was 1.44 TCDI/10,000 patient-days. Predisposing factors included solid organ neoplasms (pre-pandemic 33.7%; pandemic 33.1%; *P*=0.36), secondary immunosuppression (pre-pandemic 31.5%; pandemic 40.6%; *P*= 0.41).

**Conclusion:**

Patients admitted experienced a significantly higher risk of acquiring toxin-producing *Clostridioides difficile* infection during the pandemic. The data also indicate an increase in mortality associated with this infection and highlight proton pump inhibitor use as a contributing factor to the higher incidence.

**Table:** 

Text box 1. Contributions to the literature
• This study provides new insight into the indirect impact of the COVID-19 pandemic on healthcare-associated infections by analysing changes in the incidence of toxin-producing Clostridioides difficile infection before and during the pandemic.
• It highlights the association between this infection and common hospital-related factors, such as proton pump inhibitor use and gastrointestinal surgery, which were linked to poorer outcomes during the pandemic period.
• The findings underscore the importance of maintaining effective infection prevention measures even during public health emergencies.
• By reporting incidence rates and trends from 2017 to 2022, the study offers practical epidemiological benchmarks to support Clostridioides difficile infection surveillance and prevention planning during periods of healthcare system strain.

## Background

In 2020, the World Health Organization (WHO) declared SARS-CoV-2 a global public health emergency [1, 2]. Healthcare systems were forced to restructure their healthcare activity, resulting in the widespread implementation of infection control measures [3–5].

During the pandemic, non-pharmacological interventions were recommended to mitigate viral transmission [6, 7]. These included physical distancing, minimizing contact duration, hand hygiene, and frequent cleaning and disinfection of environments. Their effectiveness was further enhanced when implemented in combination [1, 2, 4].

Toxin-producing *Clostridioides difficile* infection (TCDI) is one of the most common causes of healthcare-associated diarrhoea in hospitalized patients [8, 9]. Despite longstanding recommendations for surveillance, evidence regarding the incidence of TCDI during the pandemic remains inconsistent. Some studies report increased incidence, although often without stratifying by hospitalization and status, which limits the ability to distinguish between hospital-onset and community-acquired cases [6, 10].

Efforts to control TCDI aim primarily to reduce contact-based transmission, the main mechanism by which the pathogen via spores are spread from symptomatic or asymptomatic individuals via healthcare personnel or contaminated surfaces. Known risk factors include advanced age (> 65 years), immunosuppression, and exposure to antibiotic or acid-suppressive therapies [11]. Age-related physiological changes, including reduced gastric acidity and alterations in gut microbiota composition, may further increase susceptibility to CDI in older adults [12]. Clinical manifestations range from asymptomatic colonization to fulminant colitis, depending largely on the host´s immune status [13]. Key preventive strategies include contact precautions, strict hand hygiene, and antim*i*crobial stewardship. Complications associated with TCDI may include hospital readmissions, surgical interventions, and death [14, 15].

In Spain, mortality attributable to hospital-acquired TCDI is estimated at 17.1 episodes per 10,000 hospital admissions [16]. The absence of a nationwide surveillance system in Spain complicates efforts to accurately assess the burden of this infection and its impact on patient outcomes [17, 18].

Previous studies have reported conflicting results regarding the impact of the COVID-19 pandemic on the incidence of *Clostridioides difficile* infection (CDI) rates. Some studies observed a decline in rates, attributing it to enhanced infection control practices. For instance, a national sample of hospital systems in the United States reported a reduction in CDI prevalence during the COVID-19 pandemic [19]. In contrast, other studies noted increases [20], potentially due to expanded use of broad-spectrum antibiotics and reduced diagnostic testing. A tertiary hospital study in northern Greece found a significant rise in CDI cases during the pandemic, while research from a Veterans Affairs hospital increased antibiotic use for pneumonia to rising CDI rates. Similar findings were reported in a single-center study from New York, which also documented increased CDI rates during the pandemic period (Zouridis et al., 2023) [20]. Many of these studies focused exclusively on general population or COVID-19-positive patients, with limited evaluation of trends among all hospitalized patients. Furthermore, data comparing both pre-pandemic periods and pandemic periods remain scarce [21].

The aim of this study is to evaluate in our tertiary hospital the incidence of nosocomial TCDI during the pandemic and to compare it with the pre-pandemic period. Additionally, the study seeks to identify periods of highest risk, explore associations with established risk factors, and investigate potentially associated with TCDI-related complications, such as hospital readmission, surgical intervention, or mortality.

## Materials and methods

### Population under study

The study was conducted in a northwestern region of Spain, specifically in Galicia (A Coruña). The A Coruña health area serves a population of approximately 550,000 individuals, with an overall average age of 60.3 years (compared to 58.0 years nationally in Spain) and a global aging index of 161.14 (compared to 133.46 nationally) [22]. Nearly 100% of the population is covered by the Galician Health System (SERGAS), which operates within the Spanish National Health System and is predominantly funded through taxation.

The University Hospital of A Coruña serves as the reference hospital for the area, classified as a tertiary care center, offering high-complexity procedures and care. The hospital has a capacity of 1,416 beds, with an average length of stay of 9.34 days and an annual occupancy rate of 79.2%, according to the 2022 Annual Report of the A Coruña and Cee Health Area [22].

### Study design and case identification

On March 11, 2020, the World Health Organization declared the COVID-19 outbreak a global pandemic. In Spain, a state of alarm was declared on March 14, 2020, in response to the public health emergency caused by the COVID-19 pandemic [1]. We conducted a retrospective observational cohort study using data from the Preventive Medicine Service and Public Health database of A Coruña University Hospital, covering the period from January 1, 2017 to December 31, 2022.

An active search was carried out for all microbiologically confirmed cases of TCDI within the health area of A Coruña, as validated by the microbiology department and documented in the database.

The Preventive Medicine and Public Health Service at A Coruña University Hospital routinely reviews all positive microbiological results for toxin-producing *Clostridioides difficile* (TCD) within the hospital’s active surveillance system. Data collection and classification of TCDI cases were performed by professionals with expertise in infection prevention and control.

### Microbiological diagnosis

**Fig. 1:**
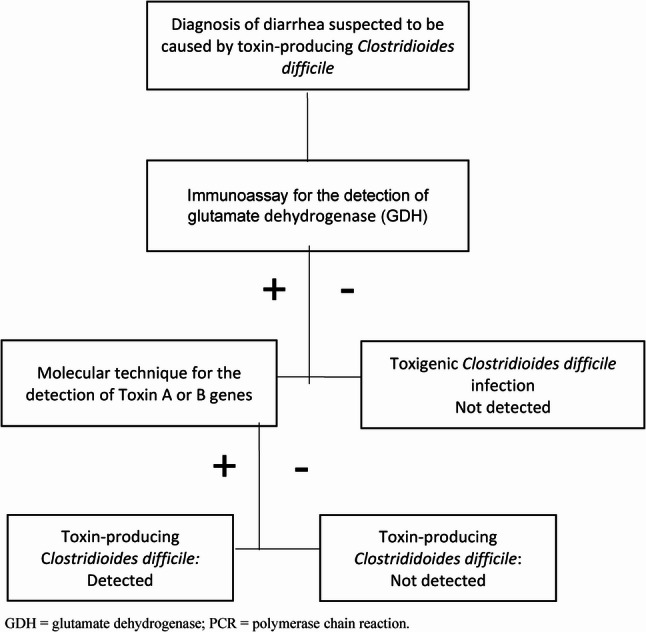
Diagnostic workflow for toxin-producing *Clostridioides difficile* infection at A Coruña University Hospital, 2017–2022. GDH = glutamate dehydrogenase; PCR = polymerase chain reaction

The diagnosis of TCDI is based initially on the presence of a positive TCD antigen in stool samples, as outlined in the diagnostic algorithm presented in Fig. 1. It is important to note that laboratory tests for TCD cannot distinguish between clinical infection and asymptomatic colonization. Therefore, a confirmed diagnosis of TCDI required microbiological detection of toxin production by the bacterium in patients with suspected TCD diarrhoea (≥ 3 unformed stools within 24 h) and no other apparent cause. Laboratory testing was conducted only for patients presenting with non-formed stool diarrhoea, classified as levels 5 to 7 on the Bristol Stool Scale [23].

As a screening method, stool samples were tested using an enzyme-linked immunoassay (ELISA) was used to detect glutamate dehydrogenase (GDH), given high diagnostic sensitivity [24]. Samples with a positive GDH result were subsequently tested using a multiplex real-time PCR assay that detects the toxins genes A and/or B, as well as the binary toxin genes (A/B), which served as confirmatory testing [25] (Fig. 1).

Molecular confirmation was performed using a real-time polymerase chain reaction (RT-PCR) assay targeting the tcdA and tcdB genes (GeneXpert^®^*Clostridioides difficile* PCR assay, Cepheid Inc.)

## Case classification

Medical records of positive cases were reviewed to identify those with symptom onset meeting the criteria for hospital-acquired infection.

Hospital-onset (nosocomial) TCDI was defined as symptom onset occurring ≥ 48 h after hospital admission or within 8 weeks after discharge from any healthcare facility, in accordance with ECDC recommendations [26]. Episodes occurring > 8 and ≤ 12 weeks after discharge were classified as community-onset, as they may still relate to recent but not immediate healthcare exposure.

Episodes occurring > 12 weeks after discharge were considered unrelated to recent healthcare exposure and were excluded from nosocomial classification.

Short hospital stays (< 24 h), such as outpatient surgery, emergency care visits, dialysis programs, or day-hospital procedures, were excluded.

Environmental cleaning in rooms of patients with suspected or confirmed *C. difficile* followed the hospital’s standard infection-control protocol, which includes the use of high-concentration chlorine-based disinfectants (sodium hypochlorite at a 1:10 dilution) for routine and terminal cleaning after patient discharge.

### Study periods and context

TCDI cases were categorized based on their occurrence relative to the SARS-CoV-2 pandemic, with two defined periods: pre-pandemic (2017–2019) and pandemic (2020–2022). During the pandemic, additional specific recommendations for cleaning and disinfection, as well as airborne and contact transmission precautions, were implemented alongside existing protocols for microorganism transmission prevention, hand hygiene, and environmental disinfection. All patients presenting with respiratory symptoms underwent microbiological testing for SARS-CoV-2 via polymerase chain reaction (PCR).

### Study size

We included all patients who met the diagnostic criteria for hospital-acquired toxin-producing *Clostridioides difficile* infection in this retrospective cohort study. No a priori sample size calculation was conducted, as all eligible cases recorded between January 1, 2017 and December, 31, 2022, were included to maximize statistical power and ensure representativeness of the population.

The data collection was carried out over the eight months following the institutional authorization of the project by hospital management (August 24, 2022) from September 1, 2022, to March 2023. Data for all study variables were extracted from the hospital´s electronic medical records system. TCDI diagnosis was based on compatible clinical symptoms and confirmed following a two-step laboratory algorithm: initial glutamate dehydrogenase (GDH) antigen detection by ELISA, followed by real-time PCR amplification of the tcdA and tcdB toxin genes for confirmation. No standalone toxin A/B immunoassay was used during the study period. Diagnosis criteria and methods remained consistent throughout the study period.

### Patient-related variables and microbiological outcomes

The primary outcome was the incidence of hospital-acquired TCDI, defined by established clinical and microbiological criteria: presence of diarrhoea (≥ 3 unformed stools within 24 h) and a positive stool test for *Clostridioides difficile* toxins.

We analyzed a comprehensive set of patient-related variables that could serve as exposures or risk factors for acquiring nosocomial TCDI. These included demographic characteristics (age, sex), clinical history (prior antibiotic use within 30 days, immunosuppression status, antiulcer therapy), and recent surgical history of gastrointestinal surgery within 30 days prior to diagnosis.

Important comorbidities and baseline health status were also considered potential as potential confounders or effect modifiers. Additionally, co-infection with SARS-CoV-2 was assessed during the pandemic period.

This integrated approach allowed us to evaluate both the combined and individual effects of demographic factors, medical history, pharmacological treatments, and comorbidities on the risk of TCDI, ensuring a thorough analysis of factors contributing to infection risk during the pre-pandemic and pandemic periods.

Data collected included patient demographics (sex, date of birth), clinical information (admission date, positive culture date, and recurrence of TCDI), as well as the classification of the case (new or recurrent).

Recurrent TCDI cases were defined as relapse or reinfection, as it is not practically feasible to differentiate between recurrence caused by the same strain or reinfection by a different strain. Episodes occurring more than eight weeks after a prior TCDI episode were classified as new cases.

### Risk variables and data collection methods

#### Morbidities related to CDI

The study recorded comorbidities associated with TCDI, including diabetes mellitus, renal disease, oncohematologic conditions, solid organ tumors, chemotherapy, and immunosuppression (primary or secondary). The McCabe scale was used to evaluate patients’ baseline clinical severity and life expectancy, categorized into three groups:

- Non-fatal disease: Life expectancy > 5 years.
- Late fatal disease: Life expectancy between 1 and 4 years.
- Rapidly fatal disease: Life expectancy < 1 year.
- Comorbidities, SARS-CoV-2 infection, and McCabe scale classifications were documented following criteria from the Spanish Prevalence Study of Nosocomial Healthcare-Associated Infections (EPINE-EPPS), endorsed by the European Centre for Disease Prevention and Control (ECDC) [8, 23, 27].

### Surgical interventions and pharmacological treatments

Data were collected on surgical interventions (date of intervention and type) and pharmacological treatment (antibiotic therapy and antiulcer therapy).

Surgical intervention: surgeries performed in the 30 days prior to CDI diagnosis. They were classified as “gastrointestinal” and “other”.

Antibiotic therapy: Antibiotics administered in the 30 days preceding the TCDI episode were documented. Patients receiving multiple antibiotic groups were included in all relevant categories. Antibiotics were classified into pharmacological groups (*c*arbapenems; cephalosporins; penicillins; other beta-lactams; quinolones; aminoglycosides; macrolides; oxazolidinones).

Antiulcer Therapy: Antiulcer treatments administered immediately prior to CDI diagnosis were documented, categorized into therapeutic groups: antacids; proton pump inhibitors (PPIs); H2 blockers; prostaglandins. Antiulcer treatments administered immediately prior to CDI diagnosis were documented. ‘Immediately prior’ was defined as any administration of proton pump inhibitors, H2-receptor antagonists, prostaglandin analogues, or antacids within the 7 days preceding CDI diagnosis.

Complications Derived from TCDI: complications were defined as:

- Readmission: Hospitalization due to CDI-related issues.
- Surgical Intervention:. This category included procedures required to manage TCDI-related complications. Fecal microbiota transplantation was not included, as no patients underwent this procedure during the study period.
- Death: Death with a demonstrable or contributory causal relationship to TCDI (either confirmed or suspected), as defined by the ECDC.
- Treatment Prescribed for TCDI: vancomycin and/or metronidazole; fidaxomicin.

### Statistical analysis

A descriptive analysis was conducted to summarize the key characteristics of the data. Categorical variables were expressed as frequencies and percentages, while continuous variables were summarized using means and medians with corresponding 95% confidence intervals (CIs).

#### Group comparisons and association testing

The association between categorical variables were assessed using the Chi-square test. Differences in medians were assessed using the Mann-Whitney U test, while comparisons of means between two groups were performed using Student’s t-test. Proportions were compared using the Z-test for independent proportions. A significance level of *P*<0.05 was considered statistically significant.

#### Incidence and risk estimation

The incidence rate of nosocomial TCDI was calculated for each study period (pre-pandemic vs. pandemic). Relative risk (RR) with 95%CI was computed to quantify the likelihood of developing TCDI during the pandemic compared to the pre-pandemic period. An RR greater than 1 indicates an increased risk associated with the pandemic period, while an RR less than 1 indicated a decreased risk.

Attributable risk, the attributable fraction among the exposed and the population attributable fraction were also calculated to estimate the potential impact of the pandemic on TCDI occurrence.

Although relative risk (RR) was used to compare incidence between periods, logistic regression was employed to explore associations with specific risk factors and adjust for potential confounders, not to estimate RR directly.

#### Multivariable analysis- period association

A multivariable binary logistic regression model was constructed to identify factors independently associated with the hospitalization period (pandemic vs. pre-pandemic), used here as the dependent variable (1 = pandemic [2020–2022], 0 = pre-pandemic [2017–2019]). Covariates include demographic variables (age, sex), comorbidities (e.g., diabetes mellitus, renal disease, malignancy, immunosuppression), SARS-CoV-2 infection status, recent exposure to antibiotic and proton pump inhibitors, recent surgical procedures within ≤ 30 days, and baseline clinical status (McCabe score).

Variables with *P* < 0.10 in univariate analyses or deemed clinically relevant were included in the model. Backward stepwise elimination was used to exclude non-significant variables (*P*>0.05). Model adequacy was evaluated using appropriate goodness-of-fit statistics tests. Subgroup heterogeneity was assessed to determine whether associations differed across strata.

#### Multivariate analysis- complications

A separate multivariable logistic regression analysis was performed to evaluate predictors of TCDI-related complications, defined as a composite outcome including hospital readmission, surgery, or death. Independent variables were coded dichotomously (“0” for “No” and “1” for “Yes.”). The model was built using forward stepwise selection. Mixed-effects models were considered to account for center-level variability. Heterogeneity tests were conducted to assess consistency of associations across subgroups and validate the robustness of findings.

### Software

All statistical analyses were performed using the STATA v17 statistical software package.

## Results

### General characteristics of the study population

During the study period, a total of 125,071 hospital admissions were recorded in the pre-pandemic years (2017–2019), compared to 116,884 admissions in the pandemic years (2020–2022), reflecting a 6.5% reduction in hospital admissions the pandemic. The total number of inpatient bed-days also decreased slightly during the pandemic: 2017–2019: 1,156,522 inpatient bed-days and 2020–2022: 1,112,661 inpatient bed-days.

A statistically significant association was found between age range and the pandemic period (χ² = 15.61; *P* = 0.01). In the pre-pandemic period, 69.7% of cases occurred in patients aged > 65 years, compared to 64.4% in the pandemic. The proportion of cases patients > 45 years differed significantly between the two periods (*P* = 0.01; 95%CI: 0.16–0.19), with 85.4% of cases in pre-pandemic and 95.6% in pandemic.

The median age of TCDI cases was similar between periods: 72.1 years [IQR 57.7–83.2] pre-pandemic and 73.1 years [IQR 59.5–82.2] during the pandemic (*P* = 0.62) (Table 1).

**Table 1:** Demographic and clinical characteristics of patients with hospital-acquired toxin-producing *Clostridioides difficile* infection at A Coruña University Hospital (2017–2022)

	Pre-pandemic (*n* = 89)	Pandemic (*n* = 160)		
Age				
Median (IQR)	72.1 (57.7–83.2)	73.1 (59.5–82.2)	Mann–Whittney Test = 0.62	
Age (SD)	66.2 (22.5)	70.2 (17.4)	68.8 ± 19.5	
			chi-squared	P>|z|
Rank Age (years)	n (%)	n (%)	15.61	0.01
< 18	6(6.7%)	4 (2.5%)		0.77
18–44	7 (7.9%)	3 (1.9%)		0.72
45–64	14 (15.7%)	50 (31.3%)		0.25
65–84	51 (57.3%)	74 (46.3%)		0.23
> 85	11 (12.4%)	29 (18.1%)		0.69
Genre			5.68	0.02
Male	40 (44.9%)	97 (60.6%)		0.06
Female	49 (55.1%)	63 (39.4%)		0.09
Morbidities				
Diabetes mellitus	27 (30.3%)	52 (32.5%)	0.13	0.73
Renal insufficiency	25 (28.1%)	47 (29.4%)	0.05	0.83
Neoplasms (SOT)	30 (33.7%)	53 (33.1%)	0.86	0.36
Immunodeficiency	35 (39.3%)	75 (46.9%)	1.30	0.25
Malignant hematopathies	13 (14.6%)	(10.6%)		0.74
Primary immunodeficiencies	1 (1.1%)	1 (1.1%)		.
Secondary immunodeficiencies	28 (31.5%)	65 (40.6%)		0.41
Chemotherapy	5 (5.62%)	19 (11.88%)		0.69
Carrier multidrug-resistant microorganisms	7 (7.9%)	16 (10.0%)	1.47	0.48
Surgical intervention	16 (18.0%)	41 (25.6%)	9.78	0.02
Non-surgical	75 (84.3%)	119 (74.4%)		0.22
Non gastrointestinal surgery	5 (5.6%)	27 (16.9%)		0.53
Gastrointestinal surgery	9 (10.1%)	14 (8.8%)		0.87
Mc Cabe Scale			4.30	0.11
Non-fatal	40 (44.9%)	81 (50.6%)		0.63
Late fatal disease	30 (33.7%)	35 (21.9%)		0.29
Rapidly fatal	19 (21.4%)	44 (27.5%)		0.61
Death associated with TCDI	4 (4.5%)	13 (8.1%)	1.20	0.80
Treatment given for the infection			17.04	0.01
Metronidazole	20 (22.5%)	14 (8.8%)		0.29
Vancomycin	54 (60.7%)	110 (69.2%)		0.28
Metronidazole + Vancomycin	9 (10.1%)	9 (5.7%)		0.73
Fidaxomicin	2 (2.3%)	20 (12.6%)		0.67
No treatment	4 (4.5%)	7 (3.7%)		0.95
Number of patients with active SARS-CoV-2 infection in pandemic years				
	2020	2021	2022	Total
Covid-19	7 (16.7%)	2 (3.8%)	12 (18.5%)	21 (13.1%)
Non covid-19	35 (83.3%)	51 (96.2%)	53 (81.5%)	139 (86.7%)

*TCDI* toxin-producing *Clostridioides difficile* infection, *SD* Standard deviation, *IQR* Interquartile range, *SOT* solid organ tumor

Male patients represented higher proportion during the pandemic (60.6%), compared to pre-pandemic (44.9%), while females predominated in the pre-pandemic (55.1%) (χ² = 5.68; *P* = 0.02).

### Epidemiology of clostridioides difficile infection

**Fig. 2:**
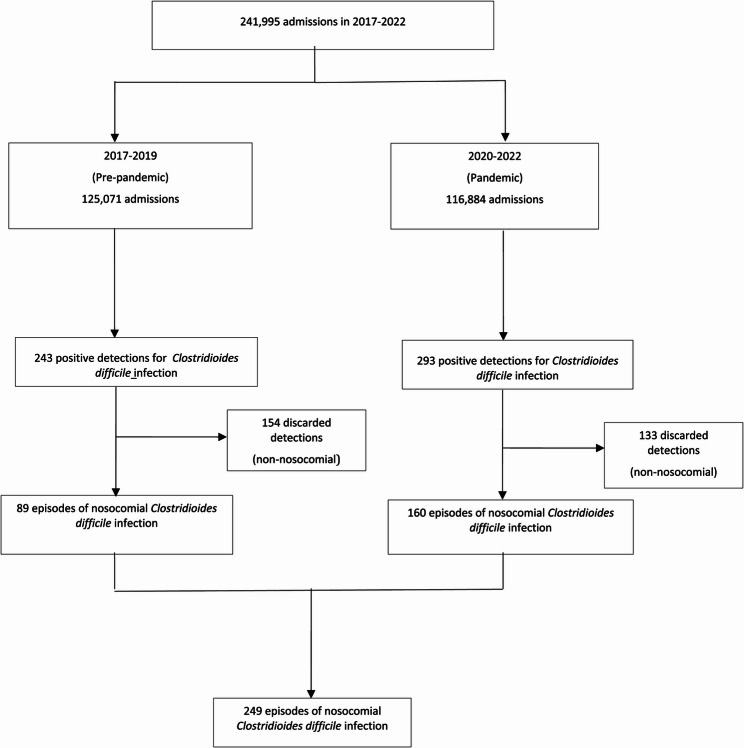
Selection flow diagram for hospital-acquired toxin-producing *Clostridioides difficile* infection cases at A Coruña University Hospital, 2017–2022

A total of 249 episodes of nosocomial TCDI were identified: 89 cases during pre-pandemic years and 160 cases during the pandemic, reflecting a 79.8% increase in incidence. The flowchart for patient selection is provided in Fig. 2.

A total of 15 recurrent CDI episodes were identified during the study period. Five recurrences occurred in the pre-pandemic years (2017–2019) and ten during the pandemic period (2020–2022). Recurrent episodes were defined as new CDI events occurring ≥ 8 weeks after a previous episode. All other CDI episodes corresponded to unique patients, meaning that no individual contributed more than one episode within the same study period unless recurrence criteria were met.

Between 2020 and 2022, 86.7% (*n* = 139) of the TCDI cases occurred in patients without active SARS-CoV-2 infection (Table 1).

### Incidence rates

The incidence density of TCDI during the pandemic years was 1.44 TCDI per 10,000 patient-days, significantly higher than the 0.77 TCDI per 10,000 patient-days recorded during the pre-pandemic period (2017–2019). The cumulative incidence was also higher during the pandemic exposure period (2020–2022), at 13.69 TCDI per 10,000 admissions, compared to 7.11 TCDI per 10,000 admissions in the pre-pandemic period. Yearly cumulative incidence rates showed an increasing trend during the pandemic years: 2020: 11.5 TCDI per 10,000 admissions; 2021: 13.7 TCDI per 10,000 admissions; 2022: 15.6 TCDI per 10,000 admissions. For comparison, the rates in the pre-pandemic years were as follows: 2017: 5,4 TCDI per 10,000 admissions; 2018: 10,3 TCDI per 10,000 admissions; 2019: 5,7 per 10,000 admissions.

No statistically significant difference was observed between the proportions of deceased patients with and without SARS-CoV-2 infection (*P* = 0.05; 95%CI: −0.30–0.05).

### Risk analysis

Compared to the pre-pandemic period, patients hospitalized during the pandemic faced a 92% greater risk of TCDI (RR = 1.92, 95%CI: 1.48–2.49; *P* = 0.001). The attributable fraction among the exposed was 48.0%, indicating that, under a causal interpretation, 48.0% of nosocomial TCDI cases in patients hospitalized during the pandemic period could be attributed to being hospitalized in 2020–2022 rather than 2017–2019 (Table 2).

**Table 2:** Incidence of hospital-acquired toxin-producing *Clostridioides difficile* infection before (2017–2019) and during the COVID-19 pandemic (2020–2022) at A Coruña university hospital

	Estimation	95% CI	*P*
Relative risk	1.92	1.48–2.49	0.01
Risk difference *1,000	0.66	0.40–0.92	
AFe *100	48.02	32.64–59.88	
PAF * 100	30.85		
Incidence CDI in the period*10,000 admissions			
Total	10.29		
Pre-pandemic	7.12		
Pandemic	13.69		
Incidence density of CDI in the period*10,000 inpatient bed days			
Total	1.10		
Pre-pandemic	0.77		
Pandemic	1.44		
CDI- mortality associated*100,000 admissions	0.70		
Pre-pandemic	3.20		
Pandemic	11.12		

Risk difference: the difference in TCDI risk between exposed (pandemic) and unexposed (pre-pandemic) groups

AFe: Attributable fraction among the exposed of nosocomial TCDI cases among patients exposed to the pandemic were attributable conditions

PAF: Population attributable fraction of all nosocomial TCDI cases in hospitalized patients were attributable to pandemic exposure

### Clinical characterístics of CDI cases

Although solid organ neoplasms (33.7% pre-pandemic; 33.1% pandemic) and secondary immunosuppression (31.5% pre-pandemic; 40.6% pandemic) were the most frequent comorbidities, their proportions did not differ significantly between the pre-pandemic and pandemic periods (Table 1). Surgical intervention history was more frequent during the pandemic (25.6%) compared to pre-pandemic (18.0%), with a significant association between surgical type and study period (χ² = 7.58; *P* = 0.05).

Additionally, the association between the McCabe risk index and the time intervals studied did not show statistical significance (χ² = 4.30; *P* = 0.11). (Table 1).

### Antibiotic therapy and drugs targeting gastric hydrochloric acid

Prior antibiotic therapy was slightly more frequent in pre-pandemic TCDI cases (86.5%) compared to pandemic cases (80.0%) (*P* = 0.20). However, the total number of antibiotics administered to TCDI patients increased by 51% during the pandemic.

The most common antibiotics groups during the pandemic were: carbapenemics: 20.6% in pre- pandemic vs. 27.1% pandemic; cephalosporins: 24.8% pre-pandemic vs. 24.9% pandemic; penicillins: 18.8% pre-pandemic vs. 13.2% pandemic.

A statistically significant difference was found in the proportion use of quinolones, which was higher in the pre-pandemic period (12.9% pre-pandemic vs. 5.1% pandemic; *P* = 0.02).

The average time from the start of antibiotic therapy to TCDI diagnosis was: 19.0 days in the pre-pandemic period (Median: 13; IQR: 3–44) and 26.6 days during the pandemic period (Median: 14; IQR: 7–24). No statistically significant differences were found between these periods.

Regarding the time from hospital admission to TCDI diagnosis, the findings were similar: Pre-pandemic: 21.8 days (Median: 13; IQR: 8–24); Pandemic: 33.2 days (Median: 14.5; IQR: 7.5–30.5). The difference between the periods was 11.42 days (95% CI: −28.8 to 5.9), with no statistically significant difference observed.

When comparing patients diagnosed during the pandemic to those in the pre-pandemic period, a statistically significant increase was observed in the use of anti-ulcer therapy (χ² = 11.39; *P* = 0.01), particularly in the use of proton pump inhibitors (PPIs), which was significantly higher during the pandemic (70.6%) than in the pre-pandemic period (51.7%) (*P* = 0.02; 95%CI: 0.02–0.36) (Table 3).

**Table 3:** Medication exposures, comorbidities, and clinical variables associated with toxin-producing *Clostridioides difficile* infection at A Coruña university hospital (2017–2022)

Antibiotic therapy given prior to diagnosis						
	Pre-pandemic		Pandemic		chi-squared	*P*>|z|
ATB*	*n* = 101		*n* = 197			0.22
Quinolones	13 (12.9%)		10 (5.1%)			0.02
Carbapenems	21 (20.8%)		52 (26,4%)			0.29
Other β-lactams	9 (8.9%)		30 (15.2%)			0.12
Cephalosporins	25 (24.8%)		49 (24.9%)			0.92
Penicillins	19 (18.8%)		26 (13.2%)			0.20
Oxazolidinones	6 (5.9%)		14 (7.1%)			0.70
Other antibiotics	8 (7.9%)		16 (8.1%)			0.95
Total patients					10.6	0.15
With antibiotic therapy	77 (86,5%)		128 (80.0%)			0.20
Non-antibiotic therapy	12 (13.5%)		32(20.0%)			0.10
Antiulcer therapy	*n* = 54		*n* = 119		11.4	0.01
PPIs	46 (51.7%)		113 (70.6%)			0.02
H2 blockers	6 (6.7%)		6 (3.8%)			0.40
Antiacids	2 (2.3%)		-			
Nº patients without anti-ulcer therapy	35 (39.4%)		47 (29.3%)			0.18

*ATB* antibiotic therapy: number of antibiotics used in all patients studied, *PPIs* proton pump inhibitors

### Treatment for TCDI

We detected a statistically significant association between the type of treatment used for TCDI and the time intervals studied (χ² = 17.04; *P* = 0.01).

The most commonly used treatment for TCDI during both periods was vancomycin: Pre-pandemic: 60.7%; Pandemic: 69.2%.

There was a notable increase in the use of fidaxomicin during the pandemic years (2020–2022): Pre-pandemic: 2.3%; Pandemic: 12.6%. However, we did not detect statistical significance between the proportions of fidaxomicin use in the two periods.

### Complications

During the years studied, 28 patients experience complication following a TCDI episode. Among them: 17 patients died and 8 were readmitted.

The variables included in the regression model were the McCabe scale, recurrence, and history of surgery. The findings were as follows: life expectancy < 1 year (McCabe scale: Rapidly fatal disease) was statistically significant associated with complications (OR 5.9; 95%CI: 2.06–16.74). Regarding surgical intervention history, gastrointestinal tract surgeries demonstrated a significant association with complications (OR 6.6, 95%CI: 2.18–20.18), as well as recurrences, where statistical significance was observed with some complication (OR 8.9, 95%CI: 2.35–33.39) (Table 4).

**Table 4:** Multivariable logistic regression analysis of risk factors associated with hospital-acquired toxin-producing *Clostridioides difficile* infection at A Coruña university hospital (2017–2022)

	*n*	OR*	*P*	95% CI
Recurrence	15	8.9	0.01	2.35–33.39
Mc Cabe Scale				
Late fatal disease	64	1.5	0.53	0.43–5.28
Rapidly fatal disease	63	5.9	0.01	2.06–16.74
Surgical intervention				
Non gastrointestinal surgery	35	1.0	0.97	0.21–4.98
Gastrointestinal surgery	22	6.6	0.01	2.18–20.18

*OR Odds ratio

We found no variables with significant results in the heterogeneity test, nor did we observe a change between the adjusted and unadjusted effects that exceeded 10%.

The TCDI-associated mortality rate was 3.20 patients/100,000 admissions in the years 2017–2019, and 11.12/100,000 in 2020–2022. (Table 2).

## Discussion

### Principal findings

This study aimed to evaluate the effect of the COVID-19 pandemic on the incidence of nosocomial *Clostridioides difficile* infection and to identify associated risk factors and complications. Our findings revealed a significant increase in TCDI incidence during the pandemic (1.44 TCDI per 10,000 patient-days), despite a reduction in overall hospital admissions. Hospitalization during the pandemic emerged as an independent risk factor for developing TCDI with a relative risk of 1.92 (95% CI: 1.48–2.49).

Although hospital admissions declined during the pandemic, the hospitalized population was more clinically complex, and patients with antibiotic exposure tended to receive a greater number of agents, which may have increased their individual susceptibility to CDI. These observations reflect the profound changes in clinical workflows and case mix at our institution during the pandemic, when reduced hospital utilization was accompanied by a higher severity of illness among those admitted. These shifts likely contributed to the increased incidence of TCDI observed in our center. The reduction in quinolone use was not accompanied by a decrease in other broad-spectrum antibiotic classes associated with CDI, such as cephalosporins and β-lactams. Additionally, although COVID-19 and CDI rarely overlapped, shifts in antibiotic prescribing practices and patient case mix during the pandemic may have indirectly contributed to the higher CDI incidence observed in our facility.

The stronger association observed between PPI use and TCDI compared with prior antibiotic exposure may reflect local prescribing practices and patient characteristics. PPI use is highly prevalent in our institution and may act as a surrogate marker for clinical complexity or severity not fully captured in our dataset. In contrast, although the overall proportion of patients exposed to antibiotics decreased during the pandemic, those who did receive antibiotics were exposed to a greater number of agents, which may have reduced the relative magnitude of this risk factor in multivariable analysis. Notably, PPI use did not decrease substantially at the start of the pandemic, which may help explain why its association with TCDI remained comparatively strong.

Several multicenter and single-center studies have reported notable reductions in CDI incidence during the early phase of the COVID-19 pandemic, largely attributed to decreased healthcare utilization and reduced exposure to healthcare-associated risk factors. These include the reports by Wright et al., Ponce-Alonso et al., Reveles et al., Merchante et al., Sipos et al., Weiner-Lastinger et al., and Evans et al. [19, 21, 28–32]. In contrast, our institution experienced an increase in TCDI during the same period. This diverge may reflect differences in patient case mix, as hospitalized patients during the pandemic in our setting were generally more clinically complex. Local antibiotic prescribing patterns may also have played a role, particularly the greater number of antibiotic agents received per exposed patient despite a reduction in the overall proportion of exposed individuals. Furthermore, variations in infection prevention practices, healthcare pressure at different pandemic stages, and the timing of local COVID-19 waves may have contributed to heterogeneous CDI trends across healthcare facilities.

During the course of the pandemic, several changes in healthcare activity and antimicrobial use occurred at our hospital that may help explain the progressive increase in TCDI observed in 2020, 2021, and 2022. In the early months of 2020, hospital occupancy dropped sharply during the first COVID-19 wave, but subsequently increased as routine clinical activity resumed and the clinical severity of hospitalized patients rose. Although the proportion of patients exposed to antibiotics decreased, those who did receive antibiotics were treated with a wider range of agents, reflecting greater therapeutic heterogeneity. At the same time, our data show a gradual increase in TCDI incidence from 11.5 cases per 10,000 admissions in 2020 to 13.7 in 2021 and 15.6 in 2022, suggesting that the combined effects of increasing patient complexity, fluctuating care pressures, and evolving antibiotic prescribing patterns may have contributed to greater susceptibility to CDI in the later phases of the pandemic.

Traditional risk factors, including antibiotic exposure and immunosuppression, remained prevalent. Notably, there was an increased use of proton pump inhibitors during the pandemic. The majority of TCDI cases occurred in patients without concurrent SARS-CoV-2 infection.

A history of gastrointestinal surgery was significantly associated with adverse outcomes. Consistent with existing literature, TCDI recurrence and baseline clinical status, as assessed by the McCabe index, were also linked to increased complications, including readmission, surgical intervention, or death. These findings underscore the effects of the pandemic on healthcare-associated infections.

### Comparison to prior work

The implementation of non-pharmacological preventive measures such as mask usage, hand hygiene, contact isolation protocols, and enhanced cleaning practices is crucial during the pandemic, especially prior to the availability of vaccines or effective treatments. In our center, hand hygiene for *Clostridioides difficile* was performed according to international guidelines, which recommend handwashing with soap and water rather than alcohol-based hand rubs, given the poor sporicidal activity of alcohol against *Clostridioides difficile* spores. Although a range of non-pharmacological infection control measures were uniformly implemented throughout the pandemic period at our center, the present study did not quantify adherence to these interventions or capture proxy metrics (e.g., consumption of disinfectants or audit scores). Consequently, while such measures may have modulated the transmission dynamics of *Clostridioides difficile*, their specific impact on nosocomial TCDI incidence remains hypothetical and warrants dedicated evaluation in future research.

Publications on TCDI trends during the pandemic present conflicting results. While some authors report an underdiagnosis of TCDI during this period [33], others have documented reductions of up to 70% in TCDI incidence among COVID-19 patients. Contrary to these findings, our data indicate a greater than 90% increased risk of CDI in our hospital during the pandemic [11].

We observed a progressive increase in the incidence of TCDIs during the years 2020, 2021, and 2022. In 2020, the year of the health alert and the widespread implementation of preventive measures at our center, the incidence of TCDIs doubled compared to the previous year. The further increases observed in 2021 and 2022 were likely influenced by the gradual relaxation of preventive measures as the pandemic progressed, particularly among patients with COVID-19.

The COVID-19 pandemic necessitated the implementation of stringent infection prevention and control (IPC) measures in healthcare settings, including enhanced hand hygiene, the use of personal protective equipment (PPE), and rigorous environmental disinfection. While these interventions were primarily designed to curb the transmission of SARS-CoV-2, they also had collateral effects on the incidence of other healthcare-associated infections, including CDI. Several studies reported an overall decline in CDI rates during the pandemic, attributing this trend to the reinforcement of IPC protocols [30, 34]. However, the effectiveness of these measures varied across different healthcare environments [21]. Critical circumstances such as increased patient volumes and resource limitations may have hindered the consistent and thorough implementation of IPC practices, thereby potentially contributing to a higher risk of hospital-acquired CDI [35, 36]. These findings suggest that although IPC measures were strengthened during the pandemic, their effectiveness in preventing CDI may have been compromised by systemic pressures and altered clinical workflows. A comprehensive understanding of these dynamics is essential to inform the development of robust, adaptable infection control strategies capable of withstanding future global health emergencies.

The dynamics of the COVID-19 pandemic throughout 2020 and 2021 were complex and characterized by successive waves, rapid viral mutations, and varying implementation of non-pharmacological measures differing in timing and intensity. Contributing factors included the increasing availability of personal protective equipment and therapeutic protocols, changes in political leadership and public attitudes, the introduction of COVID-19 vaccines, and rising vaccination coverage rates. These last two factors may have inadvertently reduced adherence to non-pharmacological control measures, leading to a relaxation of restrictions [5, 18].

Another consideration is “pandemic fatigue,” a term introduced by the World Health Organization to describe the lack of motivation and exhaustion experienced by many healthcare professionals after prolonged exposure to a serious and restrictive pandemic. This psychological burden often led to discomfort and, in some cases, abandonment of strict prevention protocols [37]. The relaxation of preventive measures likely resulted from a combination of these factors, increasing the risk of acquiring toxin-producing *Clostridioides difficile* infections.

Furthermore, as the pandemic progressed and pandemic fatigue intensified, motivation and adherence to non-COVID infection control protocols may have declined. This decline could help explain the observed increase in TCDI cases, despite the overall apparent strengthening of infection prevention and control efforts.

In line with findings from other publications, our study showed that the age group most affected by TCDI was individuals aged > 65 years in both periods [38]: pre-pandemic (69.7%) and pandemic (64.4%). TCDI diarrhoea has a particularly high incidence in the elderly population, with some studies reporting that up to 80% of cases occur in patients over 65 years of age [39]. Moreover, the incidence is approximately 10 times higher in individuals aged 60–90 years compared to younger adults.

This heightened vulnerability in the elderly can be attributed to their specific characteristics, such as higher comorbidity, immunosenescence, increased antibiotic use, multiple concurrent treatments, and frequent hospital admissions. Additionally, age-related physiological changes in the gastrointestinal tract play a significant role. These changes include reduced gastric acidity, a shift towards a predominance of aerobic flora in the gastrointestinal tract, and impaired humoral and cellular immune responses to *Clostridioides difficile*.

The absence of a significant association between the pre-pandemic and pandemic periods in our study may be explained by the ubiquity of this risk factor among patients with TCDI. Advanced age remains a predominant and consistent risk factor across both periods, irrespective of the external context.

Regarding other comorbidities, no association was observed between the percentages of McCabe Index, diabetes mellitus, or renal failure in the periods studied. While immunodeficiencies were more prevalent during the pandemic (46.9%), this difference did not reach statistical significance.

As previously discussed in relation to elderly patients (those over 65 years of age), our findings are likely explained by the fact that many of these variables are well-established risk markers for TCDI. Furthermore, several of these risk factors may have overlapped or converged across both study periods (pre-pandemic and pandemic), thus mitigating any detectable differences between them.

In our study, despite the observed increase in antibiotic use among individuals with TCDI during the pandemic years, we did not detect significant associations between the proportions of antibiotic therapy in the analyzed time periods. Recent data from the World Health Organization (WHO) reveal widespread overuse of antibiotics globally during the COVID-19 pandemic. It has been reported that although only 8% of hospitalized COVID-19 patients had bacterial co-infections warranting antibiotic therapy, approximately 75% received antibiotics “just in case” they might help. Antibiotic use varied significantly by region, ranging from 33% in the Western Pacific Region to 83% in the Eastern Mediterranean and African Regions [40].

Between 2020 and 2022, antibiotic prescriptions decreased in Europe and the Americas but increased in Africa. These studies predominantly focus on the effects of antibiotic therapy in patients actively infected with SARS-CoV-2 −24] However, there are limited studies that evaluate similar data for patients hospitalized for conditions unrelated to COVID-19 during the pandemic years.

Despite the antibiotic optimization measures implemented in our hospital, we believe that antibiotic prescribing remains consistently high, with frequent concurrent administration of multiple antimicrobial classes. This practice likely contributes to the incidence of TCDI and facilitates its subsequent spread.

We identified a significant association between the proportions of antiulcer therapy use in both study periods. With regard to acid-suppressive therapy, although the unadjusted analysis showed a significantly higher use of proton pump inhibitors during the pandemic period, this association did not persist in the multivariable logistic regression model. After adjustment for age, comorbidities, SARS-CoV-2 infection, prior antibiotic exposure, and recent surgery, PPI use did not remain an independent predictor of the pandemic period.

During 2020–2022, 70.4% of cases had a history of antiulcer therapy, reflecting an increase of approximately 20% points compared to the pre-pandemic period. Proton pump inhibitors were the most frequently utilized medications.

The suppression of gastric hydrochloric acid by antiulcer drugs has been linked to an elevated risk of infections, including viral pathogens such as SARS-CoV-2, as well as bacterial infections like TCDI. This association makes antiulcer therapy a notable risk factor for hospitalized patients [41, 42].

Additionally, other studies have associated the use of antiulcer drugs with an increased risk of TCDI, therapeutic failure, and the development of related complications [43]. However, we did not examine potential factors or conditions contributing to the increased prescription of these therapies in our hospital during the pandemic. Future research focused on optimizing the use of these drugs is warranted to mitigate associated risks.

Indiscriminate prescribing of pharmacological therapies and the relaxation of infection prevention measures are significant factors that may have influenced the rise in TCDI cases during the pandemic. Although our study did not find a statistically significant association between antibiotic use and TCDI incidence, excessive antibiotic prescription remains a critical concern, as it can disrupt gut microbiota and predispose patients to nosocomial infections. Similarly, the relaxation of infection control measures likely contributed to the spread of infections within hospitals, increasing the rate of hospital-acquired TCDI. Continuous monitoring of these factors is essential, particularly during pandemic conditions, where infection control protocols may be compromised.

Many sources report 30-day TCDI mortality rates ranging from 6% to 11% [44, 45]. All-cause data suggest that mortality among TCDI patients has increased during the COVID-19 pandemic. Some studies estimate variable mortality rates in patients with COVID-19, ranging from 8% to 80% [33, 46], often without establishing a direct association between death and TCDI. It is well-known that TCDI patients are frequently elderly and have multiple comorbidities, where the infection acts as a contributing factor to mortality [47, 48]. In our study, mortality rates during the pandemic were higher than in previous years; however, we did not find a significant association between pandemic and non-pandemic periods.

Antibiotic therapy has also been reported as a factor contributing to the increased risk of death in TCDI patients [49, 50]. Surgeries are also established risk factors for TCDI, particularly gastrointestinal procedures [21, 28]. In our study, we found no significant differences between the proportions of surgical interventions in the pre-pandemic and pandemic periods.

In our study, we observed that life expectancy < 1 year (McCabe index), gastrointestinal surgery, and TCDI recurrences were significantly associated with complications such as readmission, surgical intervention, or death. Specifically, we found an association between the response variable “complications” and factors including the McCabe index, type of surgical intervention, and recurrence. Patients with a life expectancy of less than one year demonstrated a strong association with post-infectious complications. Gastrointestinal surgery also emerged as a predisposing factor for complications. Previous studies have described that colectomies involving manipulation of the ileum are associated with an increased risk of postoperative TCDI. Our findings reinforce this association, with gastrointestinal surgeries presenting the highest risk. However, the reduced number of interventions during the pandemic may have contributed to an underestimation of these outcomes in our dataset [10].

Importantly, no CDI outbreaks were identified at our hospital during the study period. The Preventive Medicine Service conducts continuous surveillance of all microbiologically confirmed cases, and no temporal or ward-level clustering compatible with an outbreak was detected. This supports the interpretation that the progressive increase in nosocomial TCDI observed during the pandemic was not driven by discrete transmission events but more likely reflects broader changes in patient complexity, healthcare pressure, and antimicrobial exposure. Nevertheless, because a formal molecular epidemiology analysis was not performed, the possibility of small undetected clusters cannot be entirely excluded.

When examining CDI trends at our institution, we observed divergent patterns between community-onset and hospital-onset cases. Community-onset CDI decreased from 154 cases (1.23 per 1,000 admissions) in the pre-pandemic period to 133 cases (1.14 per 1,000 admissions) during the pandemic. This decline may reflect reduced healthcare-seeking behavior, decreased use of outpatient services, and lower antibiotic exposure in community settings during the pandemic, particularly during lockdown periods. In contrast, hospital-onset CDI increased, likely influenced by greater clinical complexity among hospitalized patients, higher antimicrobial heterogeneity, and increased healthcare pressure as the pandemic progressed. These opposing dynamics suggest that the pandemic differentially affected CDI epidemiology inside and outside the hospital environment.

### Strengths and limitations

This study possesses several strengths that enhance the validity of its findings. First, the inclusion of only microbiologically confirmed cases of hospital-acquired toxin-producing *Clostridioides difficil*e infection minimized misclassification bias and ensured consistency in case definition throughout the study period. Second, the analysis was based on a six-year dataset (2017–2022), covering both pre-pandemic and pandemic periods, which allowed for an adequate temporal comparison. Third, comprehensive clinical data were extracted directly from the hospital’s electronic medical records system, reducing information bias and enabling adjustment of multiple confounders in the multivariate analysis.

Nonetheless, several limitations must be acknowledged: First, the study was conducted at a single tertiary care hospital, which may influence the generalizability of the findings to other settings or populations. While the hospital´s patient demographics and clinical practices are representative of similar tertiary care centers, variations in healthcare systems, patient populations, and infection control protocol elsewhere may limit the direct applicability of the results.

Second, the absence of detailed data on community-acquired TCDI cases may have led to an underestimation of the total burden of *Clostridioides difficile* infection during the pandemic.

Third, although multiple risk factors were controlled for in the multivariate model, residual confounding from unmeasured variables- such as staffing changes, adherence to antimicrobial stewardship program, environmental factors- cannot be ruled out. Additionally, the relatively small sample size limits the statistical power and may not fully represent the phenomena in the wider study area.

Despite these limitations, the study provides valuable insights into the indirect impact of the COVID-19 pandemic on the incidence and complications of hospital-acquired TCDI. Future multicenter studies with diverse populations and prospective design are recommended to validate these findings and enhance their applicability across different healthcare settings.

Post-pandemic trends in TCDI incidence were beyond the scope of this study, which concluded in 2022. Future analyses including data from 2023 to 2024 would help determine whether the increase observed during the pandemic persisted, declined, or returned to pre-pandemic levels.

Additional limitations should also be acknowledged. First, molecular typing or ribotyping of *Clostridioides difficile* strains was not performed. Several studies have reported shifts in circulating ribotypes during the COVID-19 pandemic, including variations in the prevalence of hypervirulent strains, which may have contributed to differences in TCDI incidence. Second, although our diagnostic algorithm included the detection of the toxin genes (*tcdA*, *tcdB* and *cdtA/cdtB*), we did not undertake broader virulence profiling beyond these markers. Finally, fecal microbiota transplantation, an established alternative therapy for selected patients with recurrent CDI, is not available at our institution and therefore could not be evaluated in our cohort.

The statistical power to explore associations between COVID-19 status and TCDI was limited, as the vast majority of TCDI cases occurred in non–COVID-19 patients. Additionally, we were unable to evaluate overall PPI consumption at the hospital level, as our analysis was limited to patient-level exposure rather than institutional prescribing patterns. Therefore, we cannot determine whether a global increase in PPI use during the pandemic contributed to the observed differences.

No significant shortages of personal protective equipment, disinfectants, or infection-prevention staff occurred at our institution during the study period. During the earliest phase of the pandemic, the availability of FFP2 masks was temporarily constrained, but supply was rapidly stabilized and no documented interruptions in infection-prevention practices took place. Therefore, shortages of materials or personnel are unlikely to explain the observed increase in TCDI rates.

## Conclusion

During the pandemic years, patients admitted to our center experienced a significantly increased risk (92%) of acquiring TCDI, despite the transmission prevention and hygiene measures in place. Additionally, our findings suggest a higher mortality rate associated with TCDI during the pandemic. The use of proton pump inhibitors emerged as one of the factors linked to the increased incidence of TCDI during the pandemic.

### Future directions

Understanding the factors contributing to TCDI acquisition in hospitalized patients is crucial for designing effective preventive interventions and strategies. These measures should include robust transmission prevention protocols and the rational use of medications, particularly antibiotics and anti-ulcer therapies.

Preventive interventions must go beyond the medical community, involving all healthcare professionals, including nurses, nurse aides, and pharmacists. A collaborative approach can help optimize measures to control the transmission of *Clostridioides difficile*, ultimately reducing hospital-acquired TCDI (associated morbidity and mortality) and the economic burden on healthcare systems.

## Data Availability

The datasets generated and/or analysed during the current study are available from the corresponding author on reasonable request.

## Abbreviations

CDI: Clostridioides difficile infection
TCDI: Toxin-producing Clostridioides difficile infection
COVID-19: Coronavirus disease 2019
PPI: Proton pump inhibitor
GDH: Glutamate dehydrogenase
ELISA: Enzyme-linked immunosorbent assay
ICU: Intensive care unit
ECDC: European Centre for Disease Prevention and Control
